# A novel ferroptosis-related gene signature for overall survival prediction in patients with gastric cancer

**DOI:** 10.1038/s41598-024-53515-0

**Published:** 2024-02-23

**Authors:** Fang Wen, Fan Zhao, Wenjie Huang, Yan Liang, Ruolan Sun, Yize Lin, Weihua Zhang

**Affiliations:** 1 Nanjing University of Chinese Medicine, Nanjing, 210023 Jiangsu China; 2 College of Traditional Chinese Medicine, Nanjing University of Chinese Medicine, Nanjing, 210023 Jiangsu China; 3 Department of Oncology, Affiliated Hospital of Nanjing University of Chinese Medicine, Nanjing, 210029 Jiangsu China; 4 Clinical Laboratory Department, Hospital of the Office of the People’s Government of the Tibet Autonomous Region in Chengdu, Chengdu, 850015 Sichuan China

**Keywords:** Cancer, Computational biology and bioinformatics

## Abstract

The global diagnosis rate and mortality of gastric cancer (GC) are among the highest. Ferroptosis and iron-metabolism have a profound impact on tumor development and are closely linked to cancer treatment and patient’s prognosis. In this study, we identified six PRDEGs (prognostic ferroptosis- and iron metabolism-related differentially expressed genes) using LASSO-penalized Cox regression analysis. The TCGA cohort was used to establish a prognostic risk model, which allowed us to categorize GC patients into the high- and the low-risk groups based on the median value of the risk scores. Our study demonstrated that patients in the low-risk group had a higher probability of survival compared to those in the high-risk group. Furthermore, the low-risk group exhibited a higher tumor mutation burden (TMB) and a longer 5-year survival period when compared to the high-risk group. In summary, the prognostic risk model, based on the six genes associated with ferroptosis and iron-metabolism, performs well in predicting the prognosis of GC patients.

## Introduction

The diagnosis rate and mortality rate of gastric cancer (GC) are among the highest globally^1^. Although existing treatment methods have significantly improved the management of GC, the prognosis and 5-year survival period of advanced GC patients remain suboptimal^2^. Therefore, early diagnosis is crucial for enhancing the prognosis of GC patients^3^. GC is characterized by a complex etiology and high heterogeneity, necessitating the identification of new biomarkers with high sensitivity and specificity for early GC detection^4,5^. Additionally, due to the limited treatment options available for GC and the involvement of molecular characteristics and mechanisms in tumorigenesis, the development of new models for GC diagnosis and prognosis is necessary.

Ferroptosis, a type of programmed cell death, was initially proposed by Dixon in 2012^6^. This phenomenon is characterized by an excessive accumulation of iron ions and lipid reactive oxygen species (ROS) within the cell. Morphologically, ferroptosis is characterized by mitochondrial shrinkage and a reduction in the mitochondrial ridges^6–8^. Iron is an essential nutrient element in various cellular processes^9^. In recent years, studies have confirmed the close association between ferroptosis and cancer treatment as well as prognosis^10,11^. Hao’s research team indicated that both erastin and the overexpression of cysteine dioxygenase type 1 (CDO1) can induce ferroptosis in GC cells by inhibiting the expression of glutathione peroxidase4 (GPX4)^12^. Moreover, the tumor immune microenvironment (TIME) plays a crucial role in the pathogenesis and progression of GC^13^. Several immune cells in the TIME are closely associated with iron-metabolism and maintaining iron homeostasis^14,15^. Wang et al. revealed that interferon-gamma (IFN-γ) released by CD8+ T cells downregulates the expression of glutamate-cystine antiporter system xc-. This, in turn, inhibits cystine uptake by tumor cells, leading to lipid peroxidation and ferroptosis in tumor cells^16^. Lang and his team further demonstrated that immunotherapy sensitizes tumors to radiation therapy by promoting ferroptosis of tumor cells^17^. Therefore, targeting tumor ferroptosis mechanisms in combination with checkpoint blockade represents a potential therapeutic strategy.

In this study, we screened genes, involved in iron-metabolism and ferroptosis that are closely associated with the prognostic survival of GC patients. Subsequently, we developed a model to predict the prognostic survival of GC. Furthermore, we investigated the potential mechanisms underlying these genes and their relationship with the TIME. The findings of our study may have potential implications in predicting the 5-year survival of GC patients.

## Methods

### Data collection

Data from 375 stomach adenocarcinoma samples and 32 normal samples were retrieved from The Cancer Genome Atlas portal (TCGA, https://portal.gdc.cancer.gov/), including mRNA expression profiles, clinical parameters, and somatic mutation information. Additionally, the GSE26253 dataset was acquired from the Gene Expression Omnibus database (GEO, https://www.ncbi.nlm.nih.gov/geo). The 204 genes associated with ferroptosis and iron-metabolism were obtained from the KEGG PATHWAY Database (https://www.genome.jp/kegg/pathway.html)^18^, the Reactome Pathway Database (https://reactome.org/), the AmiGo2 database (http://amigo.geneontology.org/amigo), and related literatures. Furthermore, 318 tumor-related transcription factors were downloaded from the CISTROME database (www.cistrome.org).

### Screening the prognostic ferroptosis- and iron metabolism-related differentially expressed genes (PRDEGs) in GC

The “limma” R package was used to extract genes expression data associated with ferroptosis and iron-metabolism from the TCGA cohort. Differential expression analysis, with a false discovery rate (FDR) < 0.05, was performed to screen the differentially expressed genes (DEGs) between tumor samples and adjacent normal samples. Univariate Cox analysis was applied to assess the survival time and status of GC patients in order to identify the prognosis-related genes (PRGs) using a filter condition of *P* value < 0.05. The “venn” R package was used to identify the intersection of DEGs and PRGs, resulting in the identification of PRDEGs.

### Building and validating the prognostic risk model

We employed LASSO-penalized Cox regression analysis to construct a prognostic risk model that minimizes the risk of overfitting^19,20^. The penalty parameter (λ) for the signature was defined based on the minimum criteria obtained through tenfold cross-validation. The risk score was calculated using a specific method (${\text{Risk core = }}\sum\nolimits_{{{\text{i = }}1}}^{{\text{n}}} {\text{coefi X id}}$). Based on the median value of the risk score in the TCGA cohort, GC patients were divided into the high-risk group and the low-risk group. Subsequently, principal component analysis (PCA) and T-distribution stochastic neighbor embedding (t-SNE) analysis were performed to reduce the dimensionality of sample expression and determine whether patients in the two risk groups could be clearly distinguished. Kaplan–Meier (K–M) survival curves were used to analyze the overall survival (OS) between the two risk groups and determine the differences. Time-dependent receptor operating characteristic (ROC) curves were carried out with R package “survivalROC” to estimate the prognostic value of the risk model.

### Independence of the risk score of the prognostic risk model

We conducted univariate and multivariate Cox regression analyses to evaluate whether the risk score of the prognostic risk signature was an independent predictor of survival for GC patients. Hazard ratios (HRs) and 95% confidence intervals (CIs) were computed for the risk score and other clinical indices, where* P* < 0.05 was considered statistically significant. Nomograms, including the risk score and available clinical variables, were generated using the “rms” R package. Calibration curves of the nomograms were performed to validate if the forecast effect of the risk model aligned with the clinical reality.

### Functional enrichment analysis of differentially expressed genes based on high- and low-risk groups

The “limma” R package was utilized to obtain differentially expressed genes based on the two risk groups (DEGRGs, |log2FC| > 1, FDR < 0.05). Furthermore, we employed the R packages “clusterProfiler”, “enrichplot”, and “ggplot2” to conduct Gene Ontology (GO) and Kyoto Encyclopedia of Genes and Genomes (KEGG) analyses on these differentially expressed genes. Additionally, Gene Set Enrichment Analysis (GSEA) was employed to explore more potentially biological effects and related enrichment pathways (NOM, *P* < 0.05, FDR < 0.25).

### Analysis of the abundance of infiltrating immune cells

The comprehensive evaluation of immune-related characteristics of each sample was performed using the ssGSEA algorithm^21^, which utilized multiple immune gene sets as the core. The Cell type identification by estimating relative subsets of RNA transcripts (CIBERSORT) algorithm, an estimation method for relative subsets of RNA transcripts, was used to evaluate the degree of infiltration of 22 human immune cell subsets^22^. R software package “CIBERSORT” was employed to calculate the infiltration scores of diverse immune cells in the TIME of GC.

### Cell cultures and QPCR

Human gastric cancer cell lines (HGC-27 and MKN-45) and a human gastric mucosal epithelial cell line (GES-1) were obtained from Cell Bank, Institute of Life Sciences, Chinese Academy of Sciences Cell Bank (Shanghai, China). The HGC-27 and GES-1 cells were cultured in DMEM medium (Gibco, Grand Island, America), while MKN-45 cells were cultured in RPMI 1640 medium (Gibco, Grand Island, America), with both media supplemented with 10% fetal bovine serum (Gibco, Grand Island, America). The cells were incubated in a humidified atmosphere at 37 °C with 5% CO_2_. Total cellular RNA was extracted using the FastPure cell Total RNA Isolation Kit (Vazyme, Nanjing, China) and quantified by NanoDrop Lite spectrophotometer (Thermo Scientific). For cDNA synthesis, the total RNA underwent reverse transcription using the HiScript II Q RT SuperMix for QPCR (Quantitative real time polymerase chain reaction, Vazyme, Nanjing, China) according to the manufacturer’s instruction. QPCR was performed in triplicate on the Applied Biosystems StepOnePlus QPCR System (Thermo Fisher Scientific) using the T aq Pro Universal SYBR QPCR Master Mix (Vazyme, Nanjing, China). The relative RNA expression levels were determined, with GAPDH used as an internal control. The relative expression of each RNA was calculated using the 2^−∆∆Ct^. The primers sequences were listed in Table S1.

### Validation of the PRDEGs in clinical samples

To verify the expression of PRDEGs in clinical samples, the mRNA levels were validated by Gene Expression Profiling Interactive Analysis database (GEPIA, http://gepia.cancer-pku.cn/). The Human Protein Atlas database (http://www.proteinatlas.org) was used for the protein level.

### Statistical analysis

Perl scripts were used for sample data collation, extraction, and ID conversion. All statistical analysis were executed using R software (version 3.6.1). Wilcoxon test and Kruskal–Wallis test were used for the comparison of data between multiple groups. Pearson’s χ^2^ test or Fisher’s exact test was applied for qualitative data comparison. Survival data were analyzed using Kaplan–Meier curves and log-rank tests. Unless specified, *P* < 0.05 was considered statistically valuable, with and *P* values were two tailed.

## Results

### Determination the PRDEGs in GC

**Figure 1:**
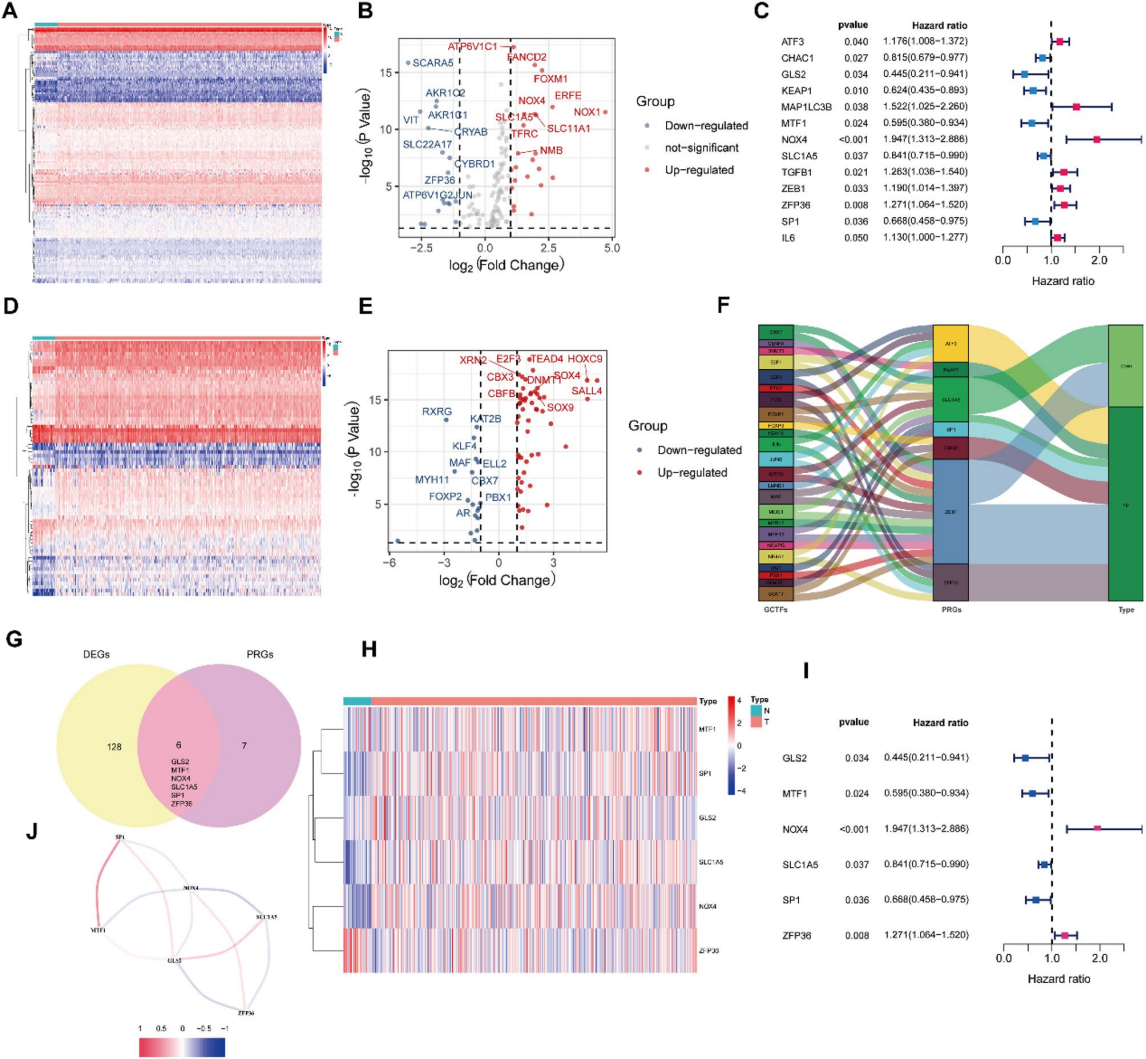
Identification the prognostic ferroptosis- and iron metabolism-related differentially expressed genes in GC. (**A**) Heatmap exhibited the expression levels of the DEGs between normal tumor and tissue. (**B**) Volcano plot of DEGs. (**C**) Forest plot showed the PRGs were associated with OS. (**D**) Heatmap exhibited the expression levels of the GCTFs between normal tumor and tissue. (**E**) Volcano plot of GCTFs. (**F**) Sankey plot of GCTFs and PRGs represented the regulatory network. (**G**) Venn diagram to determine the PRDEGs by intersecting DEGs and PRGs. (**H**) Heatmap exhibited the expression levels of the PRDEGs between normal tumor and tissue. (**I**) Forest plot showed the PRDEGs were associated with OS. (**J**) The correlation network of PRDEGs.

This study obtained a total of 134 DEGs associated with ferroptosis and iron- metabolism (Fig. 1A). The volcano plot displayed that 97 genes exhibited high expression in tumor tissues, while the remaining 37 were the opposite (Fig. 1B). Univariate Cox regression analysis was applied to filter out 13 prognostic values (PRGs) related to ferroptosis and iron-metabolism in GC patients (Fig. 1C).

By combining the previous analysis of PRGs expression and data from the ImmPort database, we screened a total of 70 differential transcription factors based on GC (GCTFs, Fig. 1D). The volcano plot revealed that 52 GCTFs were up-regulated, while 18 GCTFs were down-regulated (Fig. 1E). Furthermore, correlation analysis unveiled the regulatory network between GCTFs and PRGs. As presented in Fig. 1F, there were 37 pairs of regulatory relationships between GCTFs and PRGs, with 26 pairs being positive and 11 being negative.

To determine the six PRDEGs (GLS2, MTF1, SLC1A5, SP1, NOX4, and ZFP36), we intersected the set of 134 DEGs with the set of 13 PRGs (Fig. 1G). Figure 1H represented the differential expression of these six PRDEGs between normal and tumor tissues. Univariate Cox regression analysis confirmed that the six PRDEGs were associated with the prognosis of GC patients. Among them, NOX4 and ZFP36 were identified as high-risk genes, while GLS2, MTF1, SLC1A5, and SP1 were classified as low-risk genes (Fig. 1I). The correlation network depicted the co-expression relationship among these PRDEGs (Fig. 1J).

### Creation and verification of the prognostic risk model for GC

**Figure 2:**
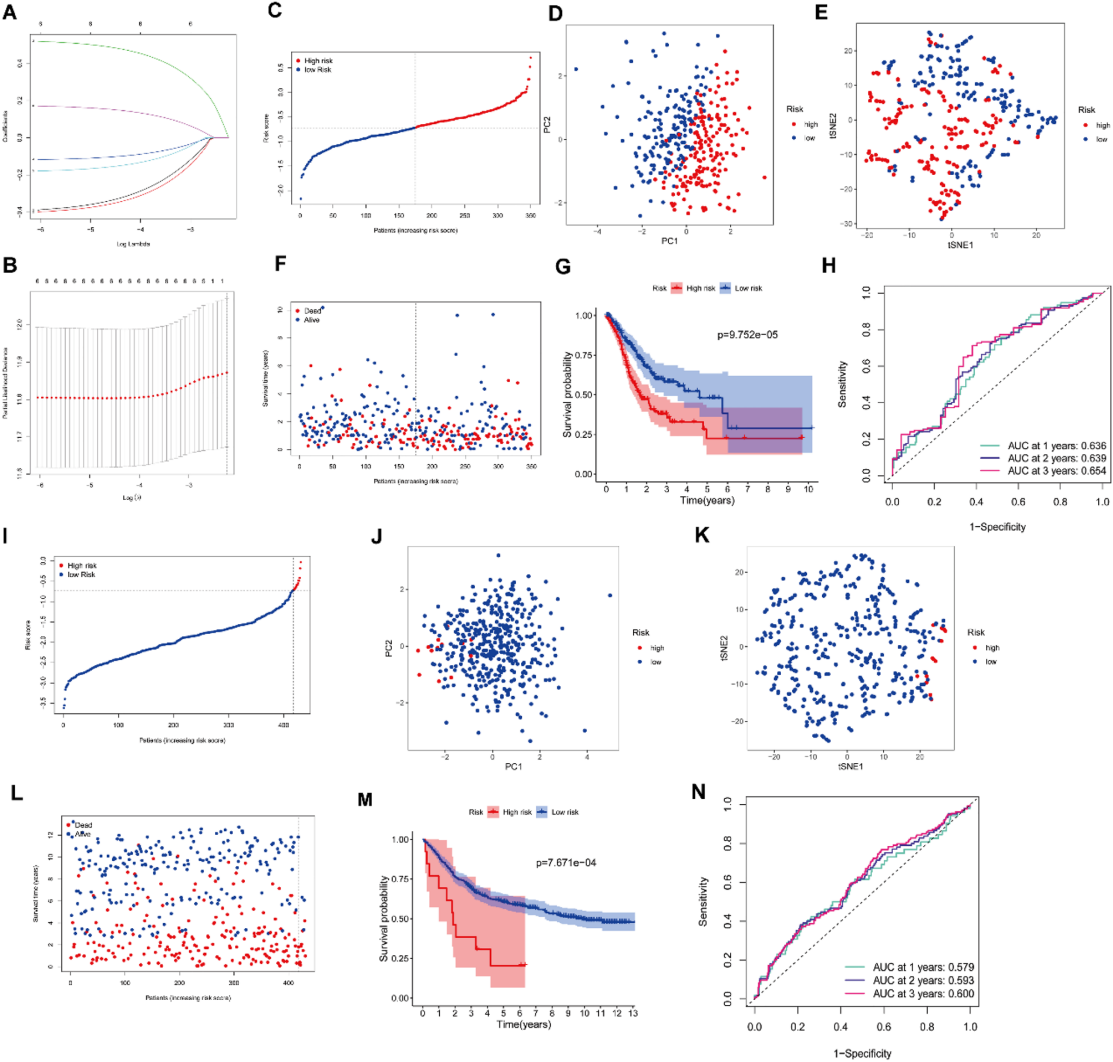
Prognostic analysis of the 6-gene prognostic risk model. (**A,B**) LASSO-penalized Cox regression analysis identified the 6-gene signature closely correlated with OS. (**C**) The distribution and the risk score median value of the TCGA cohort. (**D**) PCA analysis of the prognostic risk model in the TCGA cohort. (**E**) t-SNE analysis of the prognostic risk model in the TCGA cohort. (**F**) The distribution of the risk score and survival status in the TCGA cohort. (**G**) Survival analysis for GC patients of the different risk groups in the TCGA cohort. (**H**) AUC of time-dependent ROC curves assessed the prognostic sensitivity of the prognostic risk model in the TCGA cohort. (**I**) The distribution and the risk score median value of the GEO cohort. (**J**) PCA analysis of the prognostic risk model in the GEO cohort. (**K**) t-SNE analysis of the prognostic risk model in the GEO cohort. (**L**) The distribution of the risk score and survival status in the GEO cohort. (**M**) Survival analysis for GC patients of the different risk groups in the GEO cohort. (**N**) AUC of time-dependent ROC curves assessed the prognostic sensitivity of the prognostic risk model in the GEO cohort.

Based on the expression profile of PRDEGs and LASSO-penalized Cox regression analysis, our study has finally identified a 6-gene signature that corresponds to the optimal value of λ (Fig. 2A,B). Furthermore, we have confirmed that six PRDEGs are closely correlated with the prognosis of GC patients. The risk score is calculated as follows: risk score = (− 0.341 × expression level of GLS2) + (− 0.359 × expression level of MTF1) + (− 0.104 × expression level of SLC1A5) + (− 0.158 × expression level of SP1) + (0.489 × expression level of NOX4) + (0.154 × expression level of ZFP36).

In the TCGA cohort, 350 GC patients were divided into the high-risk group (n = 175) and the low-risk group (n = 175) based on the median value of the risk score (Fig. 2C). PCA and t-SNE analysis confirm the accuracy and distinctiveness of the grouping in the prognostic risk signature (Fig. 2D,E). Figure 2F reveals that as the risk score increases, the distribution of living patients decreased, suggesting a correlation between this risk score and the OS of patients. Moreover, the K–M survival curve demonstrates that patients in the low-risk group have a higher survival probability compared to those in the high-risk group (Fig. 2G, *P* < 0.001). The area under the curves (AUCs) of the time-dependent ROC reached 0.636 at 1 year, 0.639 at 2 years, and 0.654 at 3 years, indicating that the prognostic risk signature has a good sensitivity in predicting the survival of GC patients (Fig. 2H).

These aforementioned analysis results were validated in the GEO cohort. We divided 431 patients with GC from the GEO cohort into two groups, based on the risk score calculated from the TCGA cohort: the high-risk group consisted of 13 patients, while the low-risk group consisted of 418 patients (Fig. 2I). Similar to the results from the TCGA cohort, the PCA and t-SNE analysis show that the patients in the two groups were dispersed in opposite directions (Fig. 2J,K). Consistent with the TCGA cohort results, the patients in the high-risk group have a higher death rate and shorter OS compared to those in the low-risk group (Fig. 2L,M, *P* < 0.001). Lastly, the AUCs for the OS of GC patients were 0.579 at 1 year, 0.593 at 2 years, and 0.600 at 3 (Fig. 2N).

### The independent prognostic performance of the prognostic risk model

**Figure 3:**
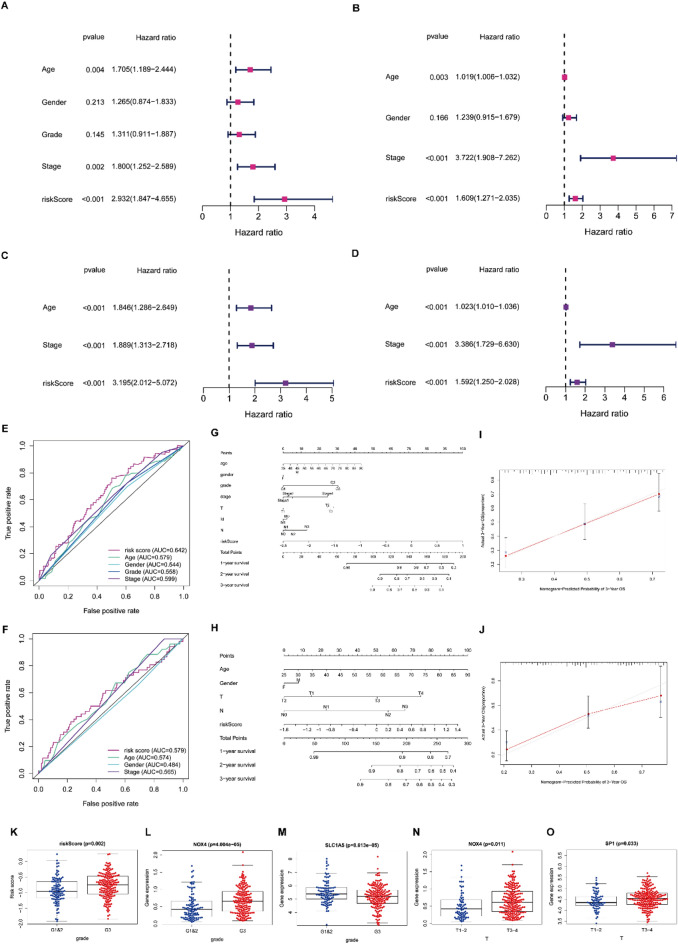
Independent prognostic performance of the prognostic risk model. (**A**) Forest plot visualizing the results of the univariate Cox regression analysis in the TCGA cohort. (**B**) The results of the univariate Cox regression analysis were verified in the GEO cohort. (**C**) Forest plot visualizing the results of the multivariate Cox regression analysis in the TCGA cohort. (**D**) The results of the multivariate Cox regression analysis were verified in the GEO cohort. (**E**) The ROC curves of the risk score and clinical indicators in TCGA cohorts. (**F**) The ROC curves of the risk score and clinical indicators in GEO cohorts. (**G**) The nomogram for predicting the survival time of GC patients at 1, 2, and 3 years in the TCGA cohort. (**H**) The nomogram for predicting the survival time of GC patients at 1, 2, and 3 years in the GEO cohort. (**I**) The calibration curves of the nomograms for survival time prediction at 3 years in the TCGA cohort. (**J**) The calibration curves of the nomograms for survival time prediction at 3-year in the GEO cohort. (**K–O**) The correlation between the PRDEGs and clinical indicators in TCGA cohort.

Univariate and multivariate Cox regression analyses were employed to determine whether the prognostic effect of the risk score on the prognosis of GC patients was independent of other available clinical indicators, including age, gender, histological grade, and TNM stage. Univariate Cox regression analyses demonstrated a signature correlation between the risk scores of the prognostic risk signature and patients’ prognosis in both cohorts (TCGA cohort: *P* < 0.001, HR 2.932, 95%, CI 1.847–4.655; GEO cohort: *P* < 0.001, HR 1.609, 95% CI 1.271–2.035) (Fig. 3A,B). Furthermore, multivariate analysis confirmed that risk score was an independent parameter for predicting patients’ prognosis (TCGA cohort: *P* < 0.001, HR 3.195, 95% CI 2.012–5.072; GEO cohort: *P* < 0.001, HR 1.592, 95% CI 1.250–2.028) (Fig. 3C,D).

To appraise the accuracy of the risk score compared to other clinical indicators, we calculated the AUCs of the ROC curves in the two cohorts. The results revealed that the risk score’s AUCs in TCGA and GEO cohorts were 0.642 and 0.579, respectively (Fig. 3E,F). Based on this, we concluded that this prognostic risk model could accurately predict the OS of GC patients. Subsequently, predictive nomograms were constructed to estimate the potential survival time of individual GC patient at 1, 2 and 3 years (Fig. 3G,H). Furthermore, calibration curves of the nomograms based on the prognostic risk signature demonstrated good consistency between predicted survival probability and actual OS at 3 years (Fig. 3I,J).

Finally, our study investigated the correlation between the prognostic risk model (including the risk score and the genes) and clinical indicators in the TCGA cohort. With an increase in the risk score and NOX4 expression, the histological grade of GC patients also increased, while the expression of SLC1A5 showed the opposite trend (Fig. 3K–M). Regarding pathological stage, elevated expression of NOX4 and SP1, was associated with higher stages (Fig. 3N,O). These results indicate an intimate connection between the abnormal expression of genes in the prognostic risk signature and the progression of GC.

### Analysis of association between risk score and TMB of GC

**Figure 4:**
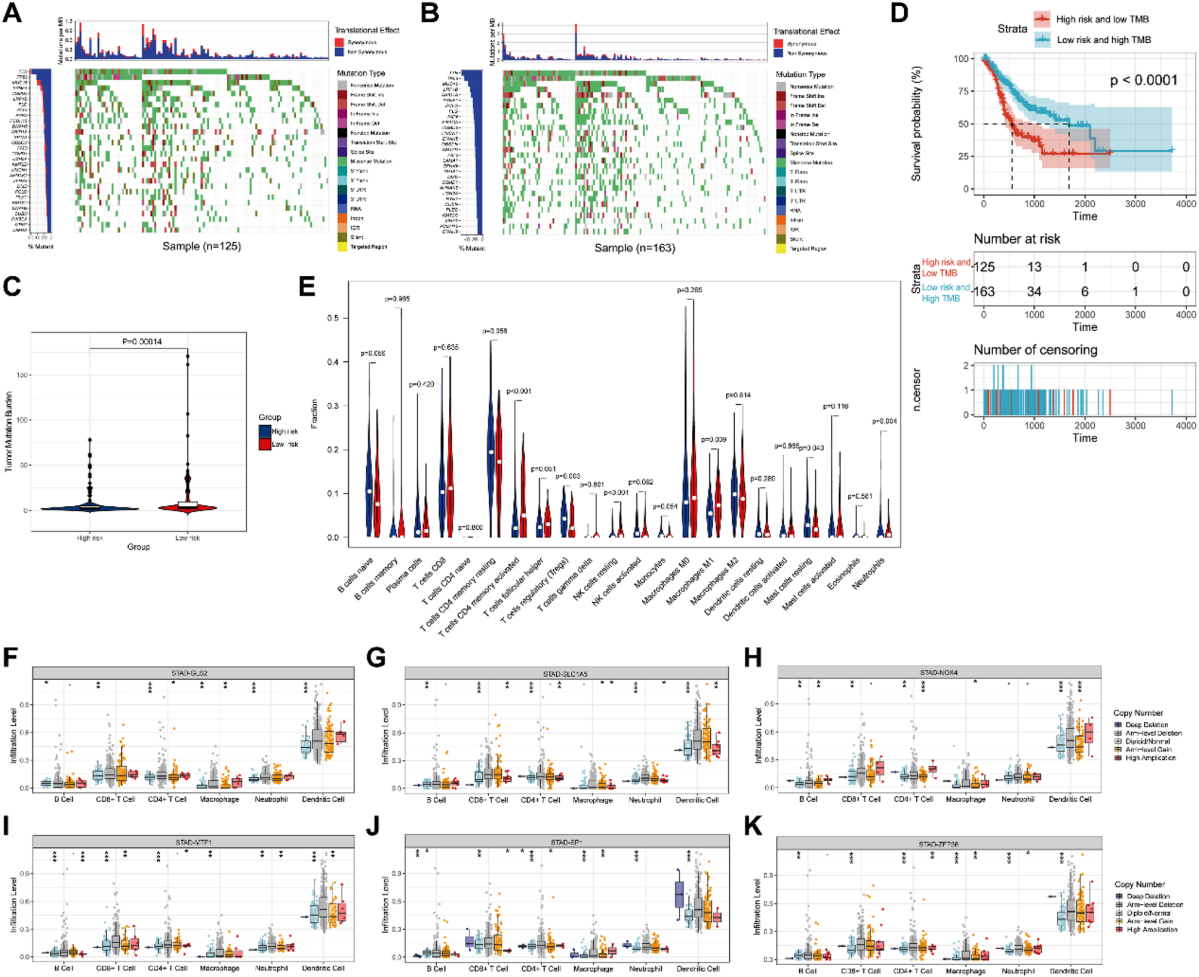
The association between risk score and TMB and the landscape of immune infiltration of TMB. (**A**) The profile of TMB in the high-risk group. (**B**) The profile of TMB in the low-risk group. (**C**) The number of TMB in the different risk groups. (**D**) K-M survival curve visualizing the differences in OS rates between the high TMB group and low TMB group. (**E**) The infiltrating levels of 22 immune cell types in different TMB groups. Blue indicated low TMB group and red indicated high TMB group. (**F–K**) Effect of somatic CNA of the 6-gene signature on the Immune Cell Infiltration. *P < 0.05, **P < 0.01, and ***P < 0.001.

The occurrence of tumors is caused by the accumulation of somatic mutations in the genes of diseased cells^23^. In this study, we first analyzed the difference in TMB between high-risk group and low-risk group of GC patients (Fig. 4A,B). Figure 4C demonstrates that the number of TMB was higher in the low-risk group compared to the high-risk group. Furthermore, patients in the low-risk group with high TMB exhibited a higher 5-year survival rate than those in the high-risk group with low TMB (Fig. 4D). TMB has emerged as a biomarker for identifying cancer patients who could benefit from immunotherapy and predicting the efficacy of immunotherapy inhibitors^23^.

Additionally, the fractions of activated CD4 memory T cells, resting natural killer (NK) cells, M1 macrophages, and neutrophils were higher in the high TMB group, while the fractions of regulatory T cells (Tregs) and resting mast cells were the opposite (*P* < 0.05, Fig. 4E).

To elucidate the underlying mechanism of the protective effects of the prognostic risk model and Immune microenvironment of GC, we further investigated the effect of somatic cell copy number alteration (CNA) of the 6-gene signature on various immune cell infiltration. CNA of the 6-gene signature significantly influenced the fractions of B cells, CD4+ T cells, CD8+ T cells, neutrophils, macrophages, and dendritic cells (DCs) in GC (Fig. 4F–K). These results indicate that the prognostic risk model plays a critical role in regulating the TIME of GC patients.

### Functional enrichment analysis

**Figure 5:**
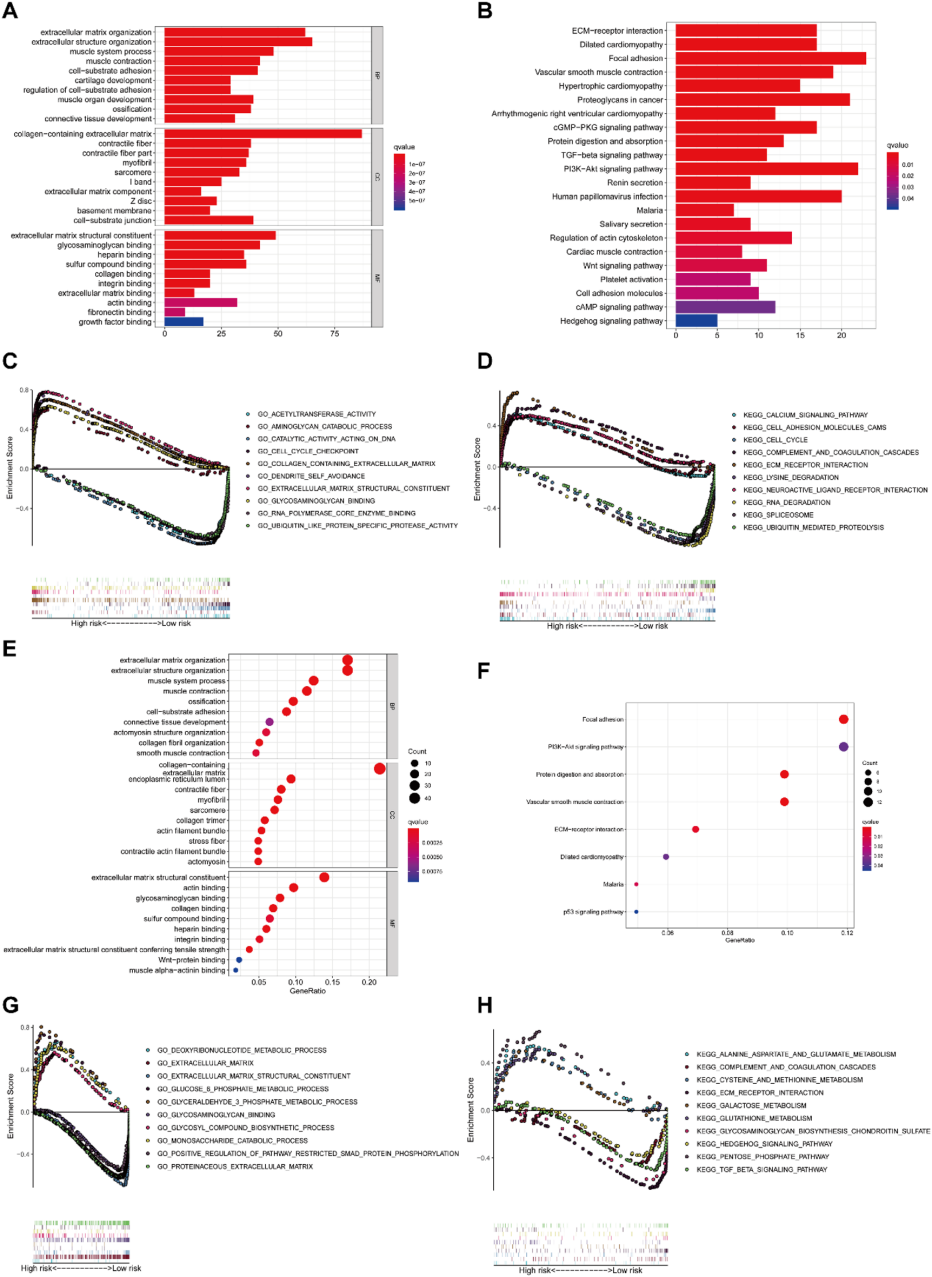
Functional enrichment analysis. (**A**) GO enrichment analysis of the DEGRGs in the TCGA cohort. The top 10 terms were displayed. (**B**) KEGG pathways analysis of the DEGRGs in the TCGA cohort. The top 10 pathways were displayed. (**C,D**) GSEA analysis on GO and KEGG in the TCGA cohort. (**E**) GO enrichment analysis of the DEGRGs in the GEO cohort. The top 10 terms were displayed. (**F**) KEGG pathways analysis of the DEGRGs in the GEO cohort. The top 10 pathways were displayed. (**G,H**) GSEA analysis on GO and KEGG in the GEO cohort.

We performed GO and KEGG analyses on the DEGRGs. In terms of GO, the DEGRGs were primarily associated with extracellular matrix organization, extracellular structure organization, and glycosaminoglycan binding in both the TCGA and GEO cohort (*P* < 0.05, Fig. 5A,E). KEGG analyses revealed that the DEGRGs were enriched in ECM-receptor interaction, PI3K-AKT signaling pathways, and protein digestion and absorption in both cohorts (*P* < 0.05, Fig. 5B,F). Subsequently, GSEA analyses were performed on GO and KEGG in the two cohorts to identify special biological processes and molecular signaling pathways. The results demonstrated that the DEGRGs from the two cohorts were primarily associated with metabolic-related biological processes (Fig. 5C,G). Furthermore, the related enrichment pathways analyses revealed that DEGRGs were mainly concentrated on lysine degradation, cysteine and methionine metabolism, glutathione metabolism, and TGF beta signaling pathway (Fig. 5D,H). These pathways are involved in ferroptosis-related biological processes and immune-related molecular functions.

### The connection between different risk groups and TIME of GC

Considering the relationship between prognosis and TIME in GC patients, we further evaluated the enrichment scores of various immune cell subsets and immune-associated effects in the two risk groups from the TCGA and GEO cohorts using ssGSEA.

**Figure 6:**
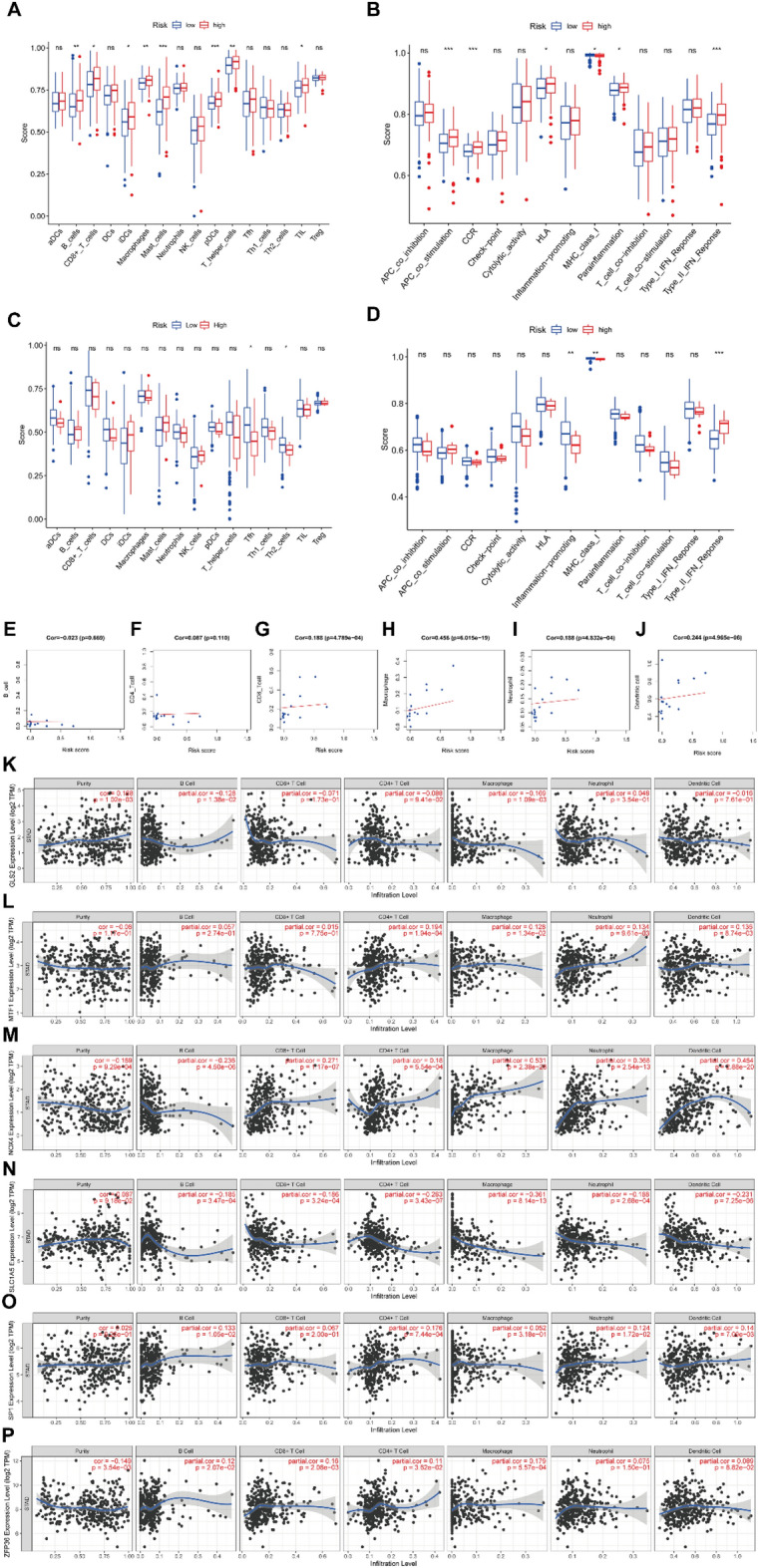
Correlation analysis of the prognostic risk model TIME of GC. (**A**) Comparison of the ssGSEA scores of diverse immune cells between the two risk groups in the TCGA cohort. (**B**) Differences of the ssGSEA scores of immune-related functions between the two risk groups in the TCGA cohort. (**C**) Comparison of the ssGSEA scores of diverse immune cells between the two risk groups in the GEO cohort. (**D**) Differences of the ssGSEA scores of immune-related functions between the two risk groups in the GEO cohort. (**E–J**) Immune cells with significant correlation with the risk score in GC patients. (**K–P**) Correlation analysis of the expression levels of PRDEGs and immune cell infiltration in GC patients. *P < 0.05, **P < 0.01, and ***P < 0.001.

For the TCGA cohort, B cells, macrophages, and plasmacytoid dendritic cells (pDCs) had higher infiltration in high-risk group. In addition, immune-related functions such as type II IFN response, APC co-stimulation, and Chemokine Receptor (CCR) were also more active in the high-risk group (Fig. 6A,B). In the GEO cohort, the scores of T follicular helper cells (Tfh), T helper type 2 (Th2) cells, inflammation-promoting factors, MHC class I, and type II IFN response showed statistically differences between the high-and low-risk groups (Fig. 6C,D).

Figure 6E–J illustrate the relationship between the prognostic risk score and various immune cells. The association between the expression of the six prognostic risk genes and immune infiltrate is shown in Fig. 6K–P.

### Validating the expression of PRDEGs in cell lines

**Figure 7:**
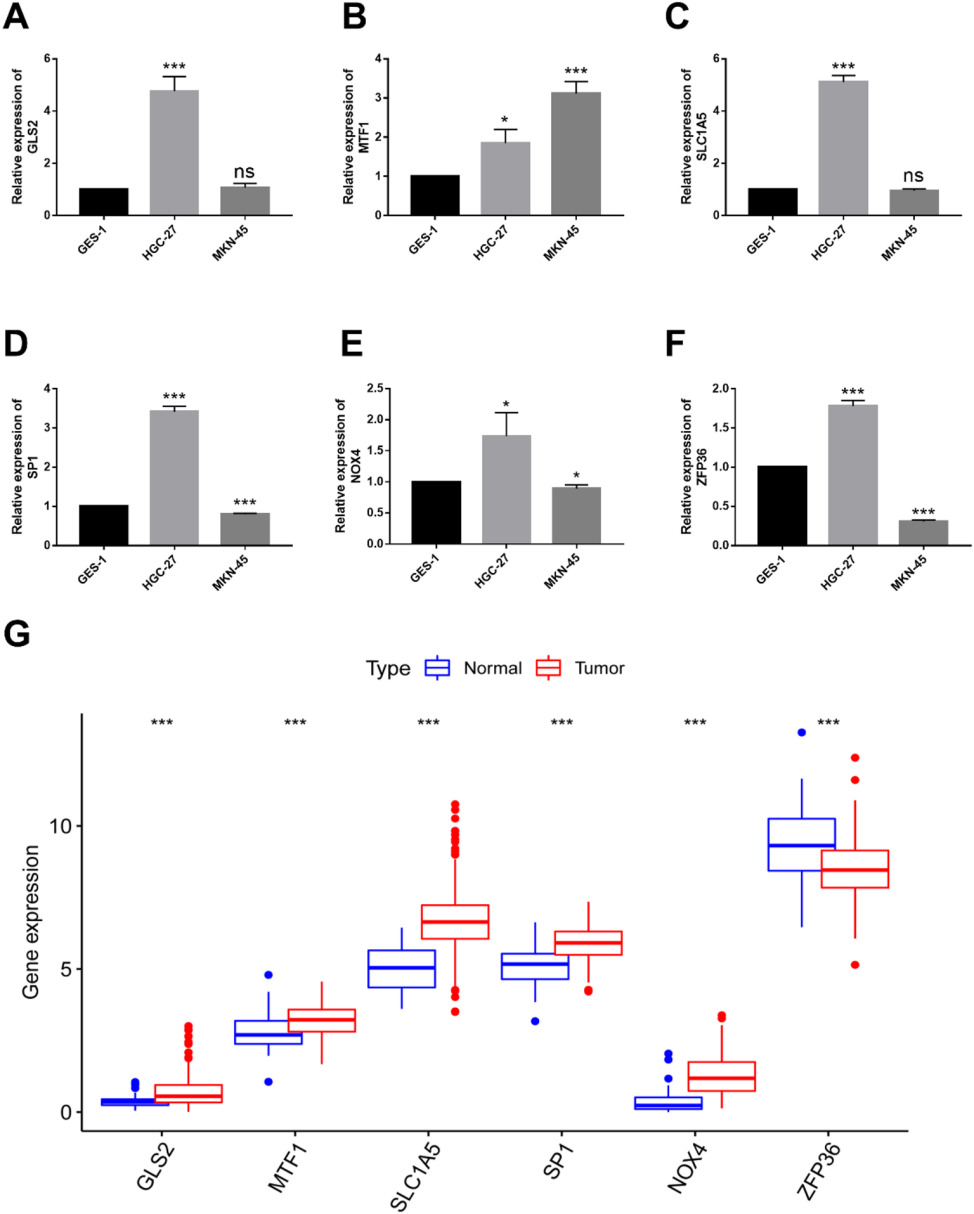
Validation the expression of the PRDEGs in cells. (**A–F**) Validation the expression of the PRDEGs in normal (GES-1) and GC cell lines. (**G**) The box plot of PRDEGs expression in TCGA. *P < 0.05, **P < 0.01, and ***P < 0.001.

The mRNA levels of PRDEGs were assessed in different GC cell lines using QPCR. The results revealed upregulation of GLS2, MTF1, SLC1A5, SP1, NOX4, and ZFP36 in HGC-27 compared to GES-1. Additionally, MTF1 showed upregulated, while SP1, NOX4, and ZFP36 were downregulated in MKN-45 compared to GES-1(Fig. 7A–F). These QPCR findings were consistent with our bioinformatics analysis results (Fig. 7G).

### Validating the expression of PRDEGs in clinical samples

**Figure 8:**
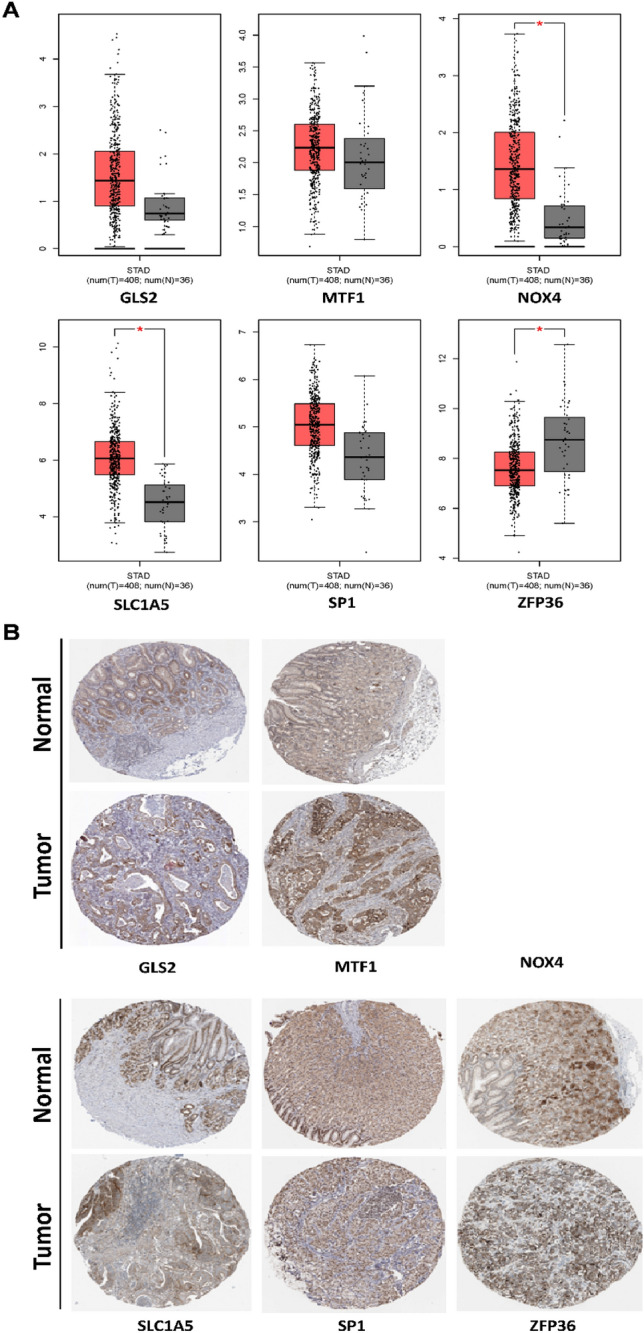
Expression of the PRDEGs. (**A**) The mRNA expression levels of the PRDEGs in GC and normal gastric tissue (*P < 0.05). Red represents GC and gray represents normal gastric tissue. (**B**) The representative protein expression of the PRDEGs in GC and normal gastric tissue.

The Gene Expression Profiling Interactive Analysis database (GEPIA, http://gepia.cancer-pku.cn/) corroborated the differences in gene expression between gastric cancers (GC) and normal gastric tissues. Boxplot showed most genes in the model had differences in GC mRNA expression compared with normal gastric tissues (Fig. 8A). Genes such as GLS2, MTF1, NOX4, SLC1A5, and SP1 were up-regulated, while ZFP36 was down-regulated. This may be related to the heterogeneity of gastric cancer. As shown in the Fig. 8B, representative protein expression was determined in the Human Protein Atlas (HPA, https://www.proteinatlas.org/). The immunohistochemistry of GC genes was positive compared with normal gastric tissues, suggesting that the protein expression was increased. However, NOX4 was not found in the database. These results are consistent with the results of mRNA expression.

## Discussion

Ferroptosis, a novel mechanism regulated by iron-dependence and lipid peroxidation, plays a crucial role in various metabolic processes and human health. It has been extensively studied in recent years, confirming its close association with the pathophysiological processes of numerous diseases, particularly in the field of oncology, which has garnered significant attention^24–28^. The emerging evidence supporting the induction of iron-mediated cell death, especially in combination with immunotherapy, offers a promising treatment approach for cancer patients^16,17^.

In this work, we first systematically to screen out 134 DEGs and 13 PRGs related to ferroptosis and iron-metabolism. Subsequently, we identified six PRDEGs, namely GLS2, MTF1, SLC1A5, SP1, NOX4, and ZFP36, through the intersection of DEGs and PRGs. A prognostic risk model was then constructed using these six PRDEGs. Our study found that this prognostic risk model exhibits strong specificity and sensitivity in predicting the prognostication of GC patients, as demonstrated through the validation in different cohorts. For details, GLS2 is an enzyme that facilitates the conversion of glutamine to glutamate, which is considered one of the 16 essential metabolic genes for tumorigenesis in functional genomics^29,30^. Recent research has revealed that GLS2 acts as a tumor suppressor gene in glioblastoma and hepatocellular carcinoma^31^. Niu et al. demonstrated that Physcion 8-*O*-β-Glucopyranoside promotes hypertrophy and inhibits tumorigenesis by inhibiting the expression of miR-103a-3p/GLS2^32^. These findings are consistent with the results of our study. MTF1 is a transcription factor responsible for regulating iron levels^33^. Its nuclear translocation protects cells from ferroptosis by reducing intercellular iron levels^34^. Chen et al. discovered that genetic depletion of MTF1 eliminated ataxia-telangiectasia mutated’s regulation of iron-regulating elements and re-sensitized cells to ferroptosis^35^. Liang and his colleagues demonstrated that over-expressed MTF1 can promote epithelial-mesenchymal transition (EMT) and ovarian tumor metastasis, making it a potential new biomarker for early diagnosis of ovarian cancer^36^. Our study discovered that low expression of MTF1 in GC patients indicates a good prognosis. SLC1A5 is a cell surface transport protein that mediates the uptake of glutamine (Gln), which is closely linked to ferroptosis^37^. Experiment have shown that miR-137 inhibits melanoma cells’ ferroptosis by targeting SLC1A5, leading to a decrease in Gln uptake and malondialdehyde (MDA) accumulation^27^. Given the crucial role of SLC1A5-regulated Gln uptake in tumor cell metabolism and ferroptosis, targeting Gln transport through the regulation of SLC1A5 could potentially serve as a therapy for cancers. Sp1 belongs to the Sp/Kruppel-like family transcription factors, known to regulate tumor occurrence and development^38–41^. Previous research has suggested that triterpenoids induced by ROS inhibit rhabdomyosarcoma cells and tumor growth by targeting Sp transcription factors (Sp1, Sp3, Sp4)^42^. Yao et al. found an association between SP1 expression and the survival time of advanced GC patients^43^. Our study further revealed that low expression of SP1 in GC patients is associated with a longer survival time. NOXs (NADPH oxidases) are membrane-binding enzyme complexes responsible for producing superoxide or hydrogen peroxide. ROS in cancer cells mainly originate from NOXs^44^. Studies have shown that inhibiting the expression of NOX4 can prevent cell ferroptosis^45^. Additionally, the increase of iron-activated Nox4 leds to excessive production of hydrogen peroxide and lipid peroxides, inducing ferroptosis in tumor cells^46^. ZFP36, a member of the tandem CCCH zinc finger proteins, has recently been identified as a new type of post-transcriptional regulator in ferroptosis^47–50^. Zhang et al.’s Studies have shown reduced transcription of ZFP36 in ferroptosis cells. Knockdown of ZFP36 induces ferroptosis, while over-expression of ZFP36 resists ferroptosis^47^. ZFP36 RNA-binding proteins (RBPs) are important immunomodulators related to cancer, playing a major role in inhibiting T cell expansion and effector function, which can be leveraged as a strategy for cancer immunotherapy^51^. In summary, the six genes included in the prognostic risk model are closely associated with iron-metabolism, lipid metabolism, oxidative stress, and immune function. In this study, these genes were highly expressed in GC tumor tissues, whereas ZFP36 showed the opposite expression pattern.

To explore the potential mechanisms of ferroptosis and iron-metabolism in GC, functional enrichment analysis and GSEA analysis of DEGRGs were conducted. The results suggested that ferroptosis in GC might be related to the metabolic process of cysteine and glutathione, along with immune-related signal pathways. To further elucidate the internal mechanism connecting ferroptosis, iron-metabolism, tumor immunity, and the prognosis of GC patients, we evaluated the differences of immune cell subsets and immune-related functions in the two groups. Our study found that the high-risk group exhibited a highly infiltration of immune-promoting cells such as B cells, macrophages, pDCs, Tfh cells, and Th2 cells, as well as increased activity of the type II IFN response (IFN-γ). Some scholars believe that the higher risk score is associated with impaired anti-tumor immunity, which may be one of the reasons for poor prognosis in high-risk patients^52^. It has been reported that high infiltration of macrophages in the TME indicates a worse prognosis for patients^53,54^. Experiments in tumor-bearing mice have showed that B cells accumulation in melanoma area could induce tumor progression, and depletion of B cells significantly improves the anti-tumor effect of Cytotoxic T lymphocytes (CTLs) and cancer^55,56^. Accumulating studies have demonstrated that pDCs have a weak ability to produce IFN-I in the TME but possess a strong capability to induce Treg cell differentiation^57–59^. Notably, pDCs in the TME not only exhibit immunosuppressive properties but also produce abnormal IFN-I^60^. IFN-γ, a major cytokine in the TME, plays a crucial role in promoting immune response^61^. However, IFN-γ not only leads to feedback suppression during the regulation of immune response but also up-regulates and induces the expression of certain immunosuppressive molecules (such as IDO1, MHCII), thus weakening the anti-tumor immune effect^62–66^. These factors may contribute to the worse prognosis observed in high-risk patients.

In recent years, numerous clinical trials and basic experiments have confirmed that the expression of immune checkpoints can serve as biomarkers for cancer patients eligible for immunotherapy. By eliminating the corresponding immunosuppression, anti-tumor immune response can be stimulated. Therefore, immune checkpoint inhibitors have been utilized to combat tumors^67,68^. It has been reported that patients with a high TMB exhibit favorable clinical efficacy when treated with immune checkpoint inhibitors^69^. In this study, we demonstrated that the low-risk and high TMB group had a better survival period, suggesting potential benefit for these patients from immune checkpoint inhibitors therapy. Additionally, our findings revealed that the high TMB group presented a higher proportion of immune-promoting cells. The CNA of the 6-gene signature significantly influenced the abundance of B cells, T cells, neutrophils, macrophages, and DCs in GC. These results further validate the regulatory role of ferroptosis and iron-metabolism on the TIME of GC.

In summary, the interplay between ferroptosis, iron-metabolism, and TIME introduces a novel research concept in the field of oncology. Investigating the hub genes associated with ferroptosis and iron-metabolism may unveil tumor antigens and enhance the immunogenicity of TME, thereby possessing significant clinical implications. Our study identified a 6-gene signature that independently predicts the prognosis of GC. This prognostic risk model exhibits strong prognostic performance, offering a new avenue for prognosis prediction in GC. However, further investigation is necessary to explore the association between these six genes and the TIME.

### Supplementary Information

Supplementary Table S1.

## Data Availability

All data were obtained from The Cancer Genome Atlas portal (TCGA, https://portal.gdc.cancer.gov/), and the Gene Expression Omnibus database (GSE26253, GEO, https://www.ncbi.nlm.nih.gov/geo).

## Abbreviations

GC: Gastric cancer
ROS: Reactive oxygen species
GPX4: Glutathioneperoxidase4
CDO1: Cysteine dioxygenase type 1
TIME: Tumor immune microenvironment
TME: Tumor microenvironment
IFN-γ: Interferon-gamma
TCGA: The Cancer Genome Atlas
GEO: Gene Expression Omnibus
PRDEGs: Prognostic ferroptosis- and iron metabolism-related differentially expressed genes
FDR: False discovery rate
DEGs: Differentially expressed genes
PRGs: Prognosis-related genes
PCA: Principal component analysis
t-SNE: T-distribution stochastic neighbor embedding
ROC: Receptor operating characteristic
HRs: Hazard ratios
CIs: Confidence intervals
GO: Gene Ontology
KEGG: Kyoto Encyclopedia of Genes and Genomes
GSEA: Gene Set Enrichment Analysis
CIBERSORT: Cell type identification by estimating relative subsets of RNA transcripts
GCTFs: Differential transcription factors based on GC
AUCs: Area under the curves
OS: Overall survival
TMB: Tumor mutation burden
CNA: Cell copy number
DCs: Dendritic cells
Tfh: T follicular helper cells
Th2: T helper type 2
CCR: Chemokine receptor
Gln: Glutamine
RBPs: RNA-binding proteins

## References

[1] Bray F (2018). Global cancer statistics 2018: GLOBOCAN estimates of incidence and mortality worldwide for 36 cancers in 185 countries. CA Cancer J. Clin..

[2] Wang J (2017). Pretreatment platelet-to-lymphocyte ratio is associated with the response to first-line chemotherapy and survival in patients with metastatic gastric cancer. J. Clin. Lab. Anal..

[3] Tan YK, Fielding JWL (2006). Early diagnosis of early gastric cancer. Eur. J. Gastroenterol. Hepatol..

[4] Chen S, Li T, Zhao Q, Xiao B, Guo J (2017). Using circular RNA hsa_circ_0000190 as a new biomarker in the diagnosis of gastric cancer. Clin. Chim. Acta.

[5] Li P (2017). Circular RNA 0000096 affects cell growth and migration in gastric cancer. Br. J. Cancer.

[6] Dixon SJ (2012). Ferroptosis: An iron-dependent form of nonapoptotic cell death. Cell.

[7] Yang WS, Stockwell BR (2008). Synthetic lethal screening identifies compounds activating iron-dependent, nonapoptotic cell death in oncogenic-RAS-harboring cancer cells. Chem. Biol..

[8] Yagoda N (2007). RAS-RAF-MEK-dependent oxidative cell death involving voltage-dependent anion channels. Nature.

[9] Andrews NC (1999). Disorders of iron metabolism. N. Engl. J. Med..

[10] Tang B (2020). The ferroptosis and iron-metabolism signature robustly predicts clinical diagnosis, prognosis and immune microenvironment for hepatocellular carcinoma. Cell Commun. Signal.

[11] Xu T (2019). Molecular mechanisms of ferroptosis and its role in cancer therapy. J. Cell. Mol. Med..

[12] Hao S (2017). Cysteine dioxygenase 1 mediates erastin-induced ferroptosis in human gastric cancer cells. Neoplasia.

[13] Zeng D (2019). Tumor microenvironment characterization in gastric cancer identifies prognostic and immunotherapeutically relevant gene signatures. Cancer Immunol. Res..

[14] Ganz T, Nemeth E (2015). Iron homeostasis in host defence and inflammation. Nat. Rev. Immunol..

[15] Wang Y, Yu L, Ding J, Chen Y (2018). Iron metabolism in cancer. Int. J. Mol. Sci..

[16] Wang W (2019). CD8+ T cells regulate tumor ferroptosis during cancer immunotherapy. Nature.

[17] Lang X (2019). Radiotherapy and immunotherapy promote tumoral lipid oxidation and ferroptosis via synergistic repression of SLC7A11. Cancer Discov..

[18] Kanehisa M, Furumichi M, Sato Y, Kawashima M, Ishiguro-Watanabe M (2023). KEGG for taxonomy-based analysis of pathways and genomes. Nucleic Acids Res..

[19] Tibshirani R (1997). The lasso method for variable selection in the Cox model. Stat. Med..

[20] Simon N, Friedman J, Hastie T, Tibshirani R (2011). Regularization paths for Cox’s proportional hazards model via coordinate descent. J. Stat. Softw..

[21] He Y, Jiang Z, Chen C, Wang X (2018). Classification of triple-negative breast cancers based on Immunogenomic profiling. J. Exp. Clin. Cancer Res..

[22] Newman AM (2015). Robust enumeration of cell subsets from tissue expression profiles. Nat. Methods.

[23] Chan TA (2019). Development of tumor mutation burden as an immunotherapy biomarker: Utility for the oncology clinic. Ann. Oncol..

[24] Zhang Z (2018). Glutathione peroxidase 4 participates in secondary brain injury through mediating ferroptosis in a rat model of intracerebral hemorrhage. Brain Res..

[25] Xie B-S (2019). Inhibition of ferroptosis attenuates tissue damage and improves long-term outcomes after traumatic brain injury in mice. CNS Neurosci. Ther..

[26] Xie Y (2017). The tumor suppressor p53 limits ferroptosis by blocking DPP4 activity. Cell Rep..

[27] Luo M (2018). miR-137 regulates ferroptosis by targeting glutamine transporter SLC1A5 in melanoma. Cell Death Differ..

[28] Van Do B (2016). Ferroptosis, a newly characterized form of cell death in Parkinson’s disease that is regulated by PKC. Neurobiol. Dis..

[29] Katt WP, Lukey MJ, Cerione RA (2017). A tale of two glutaminases: Homologous enzymes with distinct roles in tumorigenesis. Future Med. Chem..

[30] Possemato R (2011). Functional genomics reveal that the serine synthesis pathway is essential in breast cancer. Nature.

[31] Matés JM, Campos-Sandoval JA, Márquez J (2018). Glutaminase isoenzymes in the metabolic therapy of cancer. Biochim. Biophys. Acta Rev. Cancer.

[32] Niu Y, Zhang J, Tong Y, Li J, Liu B (2019). Physcion 8-O-β-glucopyranoside induced ferroptosis via regulating miR-103a-3p/GLS2 axis in gastric cancer. Life Sci..

[33] Rutherford JC, Bird AJ (2004). Metal-responsive transcription factors that regulate iron, zinc, and copper homeostasis in eukaryotic cells. Eukaryot. Cell.

[34] Lu S (2021). Ferroportin-dependent iron homeostasis protects against oxidative stress-induced nucleus pulposus cell ferroptosis and ameliorates intervertebral disc degeneration in vivo. Oxid. Med. Cell. Longev..

[35] Chen P-H (2020). Kinome screen of ferroptosis reveals a novel role of ATM in regulating iron metabolism. Cell Death Differ..

[36] Ji L (2018). Knockout of MTF1 inhibits the epithelial to mesenchymal transition in ovarian cancer cells. J. Cancer.

[37] Kanai Y (2013). The SLC1 high-affinity glutamate and neutral amino acid transporter family. Mol. Asp. Med..

[38] Yuan P (2007). Therapeutic inhibition of Sp1 expression in growing tumors by mithramycin a correlates directly with potent antiangiogenic effects on human pancreatic cancer. Cancer.

[39] Wang L (2008). Targeted inhibition of Sp1-mediated transcription for antiangiogenic therapy of metastatic human gastric cancer in orthotopic nude mouse models. Int. J. Oncol..

[40] Höcker M (1998). Sp1 and CREB mediate gastrin-dependent regulation of chromogranin A promoter activity in gastric carcinoma cells. J. Biol. Chem..

[41] Szpirer J (1991). The Sp1 transcription factor gene (SP1) and the 1,25-dihydroxyvitamin D3 receptor gene (VDR) are colocalized on human chromosome arm 12q and rat chromosome 7. Genomics.

[42] Kasiappan R (2019). Reactive oxygen species (ROS)-inducing triterpenoid inhibits rhabdomyosarcoma cell and tumor growth through targeting Sp transcription factors. Mol. Cancer Res..

[43] Yao JC (2004). Association between expression of transcription factor Sp1 and increased vascular endothelial growth factor expression, advanced stage, and poor survival in patients with resected gastric cancer. Clin. Cancer Res..

[44] Kamata T (2009). Roles of Nox1 and other Nox isoforms in cancer development. Cancer Sci..

[45] Poursaitidis I (2017). Oncogene-selective sensitivity to synchronous cell death following modulation of the amino acid nutrient cystine. Cell Rep..

[46] Wang Z (2018). Pseudolaric acid B triggers ferroptosis in glioma cells via activation of Nox4 and inhibition of xCT. Cancer Lett..

[47] Zhang Z (2020). RNA-binding protein ZFP36/TTP protects against ferroptosis by regulating autophagy signaling pathway in hepatic stellate cells. Autophagy.

[48] Tiedje C (2016). The RNA-binding protein TTP is a global post-transcriptional regulator of feedback control in inflammation. Nucleic Acids Res..

[49] Hausburg MA (2015). Post-transcriptional regulation of satellite cell quiescence by TTP-mediated mRNA decay. Elife.

[50] Masias C, Vasu S, Cataland SR (2017). None of the above: Thrombotic microangiopathy beyond TTP and HUS. Blood.

[51] Moore MJ (2018). ZFP36 RNA-binding proteins restrain T cell activation and anti-viral immunity. Elife.

[52] Liang J-Y (2020). A novel ferroptosis-related gene signature for overall survival prediction in patients with hepatocellular carcinoma. Int. J. Biol. Sci..

[53] Hu B (2020). Blockade of DC-SIGN+ tumor-associated macrophages reactivates antitumor immunity and improves immunotherapy in muscle-invasive bladder cancer. Cancer Res..

[54] Zhang Q (2019). Landscape and dynamics of single immune cells in hepatocellular carcinoma. Cell.

[55] Ganti SN, Albershardt TC, Iritani BM, Ruddell A (2015). Regulatory B cells preferentially accumulate in tumor-draining lymph nodes and promote tumor growth. Sci. Rep..

[56] Inoue S, Leitner WW, Golding B, Scott D (2006). Inhibitory effects of B cells on antitumor immunity. Cancer Res..

[57] Conrad C (2012). Plasmacytoid dendritic cells promote immunosuppression in ovarian cancer via ICOS costimulation of Foxp3(+) T-regulatory cells. Cancer Res..

[58] Labidi-Galy SI (2011). Quantitative and functional alterations of plasmacytoid dendritic cells contribute to immune tolerance in ovarian cancer. Cancer Res..

[59] Sisirak V (2012). Impaired IFN-α production by plasmacytoid dendritic cells favors regulatory T-cell expansion that may contribute to breast cancer progression. Cancer Res..

[60] Reizis B (2019). Plasmacytoid dendritic cells: Development, regulation, and function. Immunity.

[61] Ikeda H, Old LJ, Schreiber RD (2002). The roles of IFN gamma in protection against tumor development and cancer immunoediting. Cytokine Growth Factor Rev..

[62] Bald T (2014). Immune cell-poor melanomas benefit from PD-1 blockade after targeted type I IFN activation. Cancer Discov..

[63] Spranger S (2013). Up-regulation of PD-L1, IDO, and T(regs) in the melanoma tumor microenvironment is driven by CD8(+) T cells. Sci. Transl. Med..

[64] Zhao M (2007). MHC class II transactivator (CIITA) expression is upregulated in multiple myeloma cells by IFN-gamma. Mol. Immunol..

[65] Tsujisaki M (1987). Immunochemical and functional analysis of HLA class II antigens induced by recombinant immune interferon on normal epidermal melanocytes. J. Immunol..

[66] Maruhashi T (2018). LAG-3 inhibits the activation of CD4+ T cells that recognize stable pMHCII through its conformation-dependent recognition of pMHCII. Nat. Immunol..

[67] Pardoll DM (2012). The blockade of immune checkpoints in cancer immunotherapy. Nat. Rev. Cancer.

[68] Topalian SL (2017). Targeting immune checkpoints in cancer therapy. JAMA.

[69] Galuppini F (2019). Tumor mutation burden: From comprehensive mutational screening to the clinic. Cancer Cell Int..

